# Optimising recruitment and informed consent in randomised controlled trials: the development and implementation of the Quintet Recruitment Intervention (QRI)

**DOI:** 10.1186/s13063-016-1391-4

**Published:** 2016-06-08

**Authors:** Jenny L. Donovan, Leila Rooshenas, Marcus Jepson, Daisy Elliott, Julia Wade, Kerry Avery, Nicola Mills, Caroline Wilson, Sangeetha Paramasivan, Jane M. Blazeby

**Affiliations:** School of Social and Community Medicine, University of Bristol, Bristol, BS8 2PR UK; Collaboration for Leadership in Applied Health Research and Care West at University Hospitals Bristol, Bristol, BS1 2NT UK

## Abstract

**Background:**

Pragmatic randomised controlled trials (RCTs) are considered essential to determine effective interventions for routine clinical practice, but many fail to recruit participants efficiently, and some really important RCTs are not undertaken because recruitment is thought to be too difficult. The ‘QuinteT Recruitment Intervention’ (QRI) aims to facilitate informed decision making by patients about RCT participation and to increase recruitment. This paper presents the development and implementation of the QRI.

**Methods:**

The QRI developed iteratively as a complex intervention. It emerged from the National Institute for Health Research (NIHR) ProtecT trial and has been developed further in 13 RCTs. The final version of the QRI uses a combination of standard and innovative qualitative research methods with some simple quantification to understand recruitment and identify sources of difficulties.

**Results:**

The QRI has two major phases: understanding recruitment as it happens and then developing a plan of action to address identified difficulties and optimise informed consent in collaboration with the RCT chief investigator (CI) and the Clinical Trials Unit (CTU). The plan of action usually includes RCT-specific, as well as generic, aspects. The QRI can be used in two ways: it can be integrated into the feasibility/pilot or main phase of an RCT to prevent difficulties developing and optimise recruitment from the start, or it can be applied to an ongoing RCT experiencing recruitment shortfalls, with a view to rapidly improving recruitment and informed consent or gathering evidence to justify RCT closure.

**Conclusions:**

The QRI provides a flexible way of understanding recruitment difficulties and producing a plan to address them while ensuring engaged and well-informed decision making by patients. It can facilitate recruitment to the most controversial and important RCTs. QRIs are likely to be of interest to the CIs and CTUs developing proposals for ‘difficult’ RCTs or for RCTs with lower than expected recruitment and to the funding bodies wishing to promote efficient recruitment in pragmatic RCTs.

**Electronic supplementary material:**

The online version of this article (doi:10.1186/s13063-016-1391-4) contains supplementary material, which is available to authorized users.

## Background

Randomised controlled clinical trials (RCTs) are regarded as the most effective and efficient study design for the evaluation of healthcare interventions. Many RCTs have identified successful interventions that have become widespread or prevented the use of interventions thought to be effective but shown to be harmful. Consultations have shown high levels of willingness on the part of patients to take part in research [1], and patient and public involvement is now an integral part of RCT development in many countries, including the US (http://www.pcori.org) and UK (http://www.nihr.ac.uk/funding/pgfar-patient-and-public-involvement.htm). The increasing complexity and expense of contemporary clinical and public health practice demands evidence from high-quality pragmatic trials across the spectrum of health care. RCTs are being undertaken in increasing numbers to provide it. A major problem with many RCTs, however, is difficulty with participant recruitment [2]. Recruitment difficulties can lead to RCTs requiring considerable additional research resources in extensions, being underpowered or failing to be completed, or taking so long that their interventions become outdated. Reduced or restricted recruitment may also have implications for the generalisability of an RCT’s findings, particularly if only a very small percentage of eligible patients are recruited overall or from particular centres in multi-centre trials or particular groups of patients are not recruited such as members of ethnic minorities or populations with the greatest need.

Many attempts have been made to improve recruitment, documented in systematic reviews [3–5], but surprisingly few robustly evaluated or successful recruitment strategies are generalizable [6].

Recruitment difficulties are poorly understood, mostly because when they occur, chief investigators (CIs) and Clinical Trials Units (CTUs) try to do everything they can to improve recruitment, making it difficult to work out what has made a difference [7]. Research using qualitative methods has increased our understanding of recruitment, including that it is a complex process rather than an event [8], is difficult for patients because of its technical (non-lay) concepts [9–11], and provokes ‘hidden’ challenges for recruiters [12–14]. These accumulated understandings, allied with the need for greater patient participation, have enabled the development of an intervention that offers opportunities to avoid or address recruitment difficulties, even in the most challenging trials. The development of the intervention is reported here, including details of its components and dual format (for RCTs in development or underway with recruitment problems), and a formative evaluation of its implementation in several ongoing RCTs.

## Methods

### Intervention development

The development of the recruitment intervention followed an iterative and accumulative process, and while this largely mirrored guidelines now published [15–17], it did not strictly follow all the recommendations, as they were not then available. The first version of the recruitment intervention was developed in the feasibility study of an RCT that was considered difficult for recruitment: the NIHR ProtecT (Prostate cancer testing and Treatment) trial with randomisation between radical surgery, radical radiotherapy, and active monitoring for clinically localised prostate cancer. This trial started in 1999, and the recruitment was completed in 2009. The theoretical framework, the context for the intervention, and the evolution and key components of the framework have been published elsewhere [18–20]. Additional file 1 contains RCT registration details.

The initial version of the recruitment intervention was then applied through the MRC Quartet (Qualitative Research in Trials) programme in four RCTs expected to have recruitment challenges in different contexts: mental health, paediatrics, treatment for laryngeal cancer, and follow-up strategies following treatment for cancer [8]. Three RCTs completed recruitment successfully (see, for example, [21]) and the other closed with clear explication [22]. After further refinement, the near-final intervention was applied to another cancer trial, which also closed with clear reasons [23]. These six RCTs were phase III, pragmatic, unblinded trials in feasibility stages or full-scale recruitment, and all were considered challenging for recruitment because of very contrasting arms, no-treatment comparators, or controversial clinical contexts. The accumulated experience and data from these RCTs were synthesised and used to finalise the intervention, which was then applied in seven further RCTs by the QuinteT (Qualitative Research Integrated in Trials) team (http://www.bristol.ac.uk/social-community-medicine/research/groups/social-sciences-health/quintet/qri-rcts/). The intervention was named after the team: the QuinteT Recruitment Intervention (QRI).

### Research methods

The first version of the intervention applied in the ProtecT trial had used primarily qualitative research methods. The ProtecT feasibility study innovatively “embedded the randomised trial within the qualitative study” [18]. Qualitative, in-depth interviews were undertaken with the patients eligible for randomisation as well as the urologists and nurses who were undertaking recruitment appointments and were analysed using standard thematic approaches [24]. A crucial innovation was to routinely audio-record all recruitment appointments. Analysis of these interactions was instrumental in uncovering key issues that were hindering recruitment—such as the recruiters’ difficulties in explaining the ‘no-treatment’ arm and patients’ unwillingness to accept it as ‘watchful waiting’ [18]. This finding led to the detailed specification and re-naming of the option as ’active monitoring’, which better reflected its intention of enabling patients to avoid unnecessary treatment under close surveillance [18]. In time, further analysis of the recruitment appointments revealed key aspects of communication that facilitated recruitment [25] and a nuanced understanding that patient preferences, usually perceived as a barrier to recruitment, could instead be explored to facilitate enhanced informed consent and increased randomisation [26, 27].

In the application of the intervention to the four further RCTs in the Quartet Programme, however, it was clear that standard methods of qualitative data collection and analysis were too time-consuming when faced with the demands of reporting findings and acting on them to improve recruitment in ongoing RCTs [8]. Innovative applications of existing methods, and new research methods were thus developed. Targeted techniques of analysis of audio-recordings of recruitment appointments were developed and implemented [18, 25], adapted from conversation analysis [28], focusing, for example, on interactions related to specific issues such as randomisation. Simple quantification of eligible patients, patient pathways, and recruitment rates were added to assess the complexity of the recruitment process. A novel mixed-method approach, combining appointment timings and qualitative interpretation—quanti-qualitative appointment timing (Q-QAT)—was developed to identify an unbalanced presentation of the RCT information hindering recruitment [29]. Further developments to increase the rapidity of data collection and analysis, while retaining robustness, are underway.

### The finalised intervention: the QRI

The final form of the QRI uses standard and innovative qualitative research methods with simple quantification to enable speedy implementation. In the sections that follow, the findings from the development work that informed the design of the QRI are presented with a clear exposition of components of the QRI and examples of its implementation in two RCTs. Ethical approval for the research was obtained (see Additional file 1), and informed consent to participate was obtained in writing.

## Results

### Findings from the development work

Valuable lessons were learned about applying the QRI in the first six RCTs. Three RCTs had a feasibility phase because the CI had anticipated recruitment difficulties; three were undertaking main recruitment but suffered severe recruitment problems. The RCTs included a range of interventions, including drugs, surgery, radiotherapy, and social support; sometimes the RCTs included a ‘no-treatment’ comparator. Several issues arose that made collaboration difficult. The need to put new ethical processes and governance arrangements in place for the QRI when RCTs were already underway caused delays in commencing the research [8]. Although audio-recording was included in the protocol, many recruiters avoided or actively resisted taking recordings, and CIs did not always appreciate its importance or support its implementation. Recruitment pathways were often found to be complicated, with variations between centres. The time-consuming nature of the primarily qualitative research, combined with delays in starting and a lack of audio-recordings, made it difficult to implement the intervention fully [8]. Most CIs acknowledged positive contributions to recruitment from the intervention, including having clear explanations for recruitment difficulties, but it was often hard to define the precise contribution that the intervention had made. It was concluded that QRIs might best be integrated into feasibility studies and main RCTs with committed CIs (like ProtecT) [8].

A gap in the literature in relation to the perspectives of recruiters has been noted [6]. As in-depth interviews had been conducted with RCT designers and recruiters in all six RCTs as part of the development of the QRI, the findings were re-analysed and synthesised to provide insights into the perspectives of recruiters [12, 13]. In three of these RCTs, doctors and nurses were undertaking recruitment together, in two it was primarily nurses, and in one, primarily doctors. The research synthesis revealed that the recruitment process was complex and fragile, [12] with ‘clear obstacles’, defined as commonly reported organisational/logistical challenges, unexpectedly lower numbers of eligible patients, strong patient preferences for particular interventions, and patients seemingly unwilling to consider randomisation [3, 30]. In addition, the synthesis identified a number of other previously undocumented issues defined as ‘hidden challenges’, which were related to recruiters’ difficulties with key aspects of the RCT design and perceived conflicts between clinical and research roles [12], and the emotional and intellectual challenges they experienced when attempting to recruit patients [13].

The accumulated findings and experience from these studies enabled the development of the finalised QRI, described in full below.

### The QuinteT Recruitment Intervention

The premise of the QuinteT Recruitment Intervention (QRI) is simple, that is, to understand the reasons for recruitment difficulties so that steps can be taken to address them. This will ensure more efficient and effective recruitment and engaged informed consent or clear documentation of why recruitment is not possible and should cease. The aims of the QRI are thus as follows:

1. Understand the recruitment process in the RCT as designed and implemented in its clinical setting, including differences between centres in multi-centre RCTs
2. Document reported recruitment barriers, and understand the source of the difficulties—specifically whether they are ‘clear obstacles’ or ‘hidden challenges’ (see above and [12])
3. Assess the accuracy and clarity of the presentation of the RCT by recruiters and the level of engagement and informed consent achieved by participants
4. Present evidence about generic and RCT-specific recruitment difficulties to the CI, CTU, and Trial Management Group (TMG) in a timely manner to enable the production of a plan of improvement
5. To implement the plan of improvement as agreed with the CI, CTU, and TMG

**Fig. 1:**
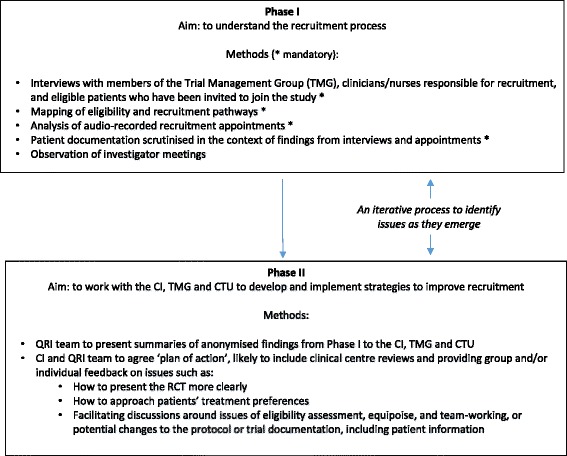
An outline of the QuinteT Recruitment Intervention (QRI) phases

A QRI is undertaken in two main phases (see Fig. 1 for details):

### QRI phase I: understanding recruitment

The aim of phase I is to understand how the RCT protocol is operationalised in clinical centres, to assess the degree to which the RCT is integrated into the clinical service, identify the patient pathway through eligibility assessment and recruitment (including where potential participants might be ‘lost’), assess levels of informed consent, and elucidate ‘hidden challenges’ to recruitment that are emerging unwittingly through the actions or presentation of information by recruiters. There are four mandatory (essential core activities) and one optional (helpful but not essential) components, utilising standard and innovative methods of data collection and analysis undertaken by an experienced qualitative researcher:

1. In-depth interviews (mandatory)
  In-depth semi-structured interviews are conducted (and audio-recorded with consent) with the following:
  The CI and key members of the TMG involved in the design and running of the RCT. These provide an overview of their view of the design of the RCT; the evidence underpinning it; details of the intervention and control arms, terminology used, and organisational arrangements; and views about the reasons for recruitment difficulties.
  Individuals involved in presenting the RCT to patients, including principal investigators responsible for clinical centres, and clinical/nurse/research staff who undertake the assessment of the eligibility of participants or have a role in explaining or presenting the details of the RCT. The most important are those who formally obtain informed consent for randomisation. A sample of staff may need to be selected in large trials or centres (see below). The aim is to understand the recruiters’ perspective of the RCT design, evidence and protocol, and recruitment pathways and challenges.
  Patients who have declined to take part in the RCT. If recruitment is very low, patients can provide interesting insights into how the RCT has been presented by recruiters and why they did not wish to take part.
  Interviews should employ standard methods of qualitative data collection, with attention to sampling to ensure inclusion of a wide range of views, for example using standard purposive techniques to identify a range of experiences. Interviews need to be audio-recorded and analysed using constant comparison techniques with the aim of drawing out key themes representing the perspectives of those interviewed, including the identification of ‘clear obstacles’ and ‘hidden challenges’. Any data presented must be anonymised.
2. Eligibility and recruitment logs, and charts of the patient pathway (mandatory)
  A flow chart should be completed in each clinical centre to show the pathway that patients follow from eligibility through randomisation, including who they see, when they receive written information about the RCT, who assesses eligibility, and who describes the RCT to them in detail, including gaining written informed consent and completing the process of randomisation. A log of patients who are potentially eligible should also be kept and maintained by the RCT team. We have devised the SEAR framework to assist with this – collecting information on **S**creening (including those who should be considered for the RCT according to the protocol), **E**ligibility (including whether they met the protocol inclusion/exclusion criteria, and for what reason they were deemed ineligible), whether they were **A**pproached about RCT participation (if not - why), and finally whether they were **R**andomised (if not, why not and which treatment they received) (Wilson C et al.: The Screening, eligibility, approached and randomised framework for RCT recruitment, forthcoming).
  Simple counting of data collected in SEAR logs can provide useful information about the complexity of the recruitment process, and differences between centres or over time can give indications of difficulties that can be investigated further.
3. Audio-recording of recruitment appointments (mandatory)
  The most important part of the QRI is the analysis of the interactions between recruiters and potential RCT participants in the appointments where recruiters explain the design and details of the RCT, and patients decide whether or not to take part. The routine audio-recording of such appointments can provide extremely valuable information about recruitment as it actually happens—information not available directly from any other source. Time spent explaining aspects of the RCT can be documented and quantified using the Q-QAT technique, giving useful indications of the order of presentation and degree of balance between the RCT interventions, and the time the RCT is first mentioned and how long is devoted to it [29]. The audio-recordings of the interaction between recruiter and participant can be analysed using content, thematic and targeted CA techniques to elucidate reasons for imbalances in presentation, style and content of information provided by the recruiter, participation and engagement of patient, and indications of the presence and origin of ‘hidden challenges’.
4. Study documentation (mandatory)
  Patient information sheets (PIS), consent forms and the study protocol should be scrutinised in the context of findings from interviews and appointments to identify aspects that might be unclear or potentially open to misinterpretation.
5. Observations of investigator meetings (optional)
  TMG meetings, site visits and other investigator meetings may be observed and/or audio-recorded to identify how the obstacles or challenges to recruitment are being addressed.

### QRI phase II: feedback to CI/TMG and plan of action

Phase II begins with the QRI researcher presenting summaries of anonymised findings to the RCT CI and CTU staff and to the TMG (if agreed earlier), including supporting evidence. No identifiers of individuals or clinical centres will be shown in presentations or reports describing the factors that appear to be hindering recruitment. In some cases, the evidence from the QRI can provide clear reasons why recruitment to the RCT may not be achievable. In this situation, the QRI researcher will prepare a report presenting the evidence for the CI/TMG to facilitate their decision making. In most cases, a potential plan of action to improve recruitment will be proposed, based on the findings from the specific RCT but also including experience from other QRIs for more generic issues. Discussions will be held with the CI/CTU/TMG to decide on the content of the plan (see below) and responsibilities for implementation.

The plan of action is likely to include providing feedback and training to recruiters on issues such as how to present the RCT’s design and interventions more clearly to improve levels of understanding and informed consent, how to approach patients’ treatment preferences, and, perhaps, facilitating discussions around ‘hidden’ issues of eligibility assessment, equipoise, and team-working, or potential changes to the protocol or patient information and consent forms. The plan usually contains a mix of generic RCT-related issues, such as explanation of RCT procedures, and issues specific to the particular RCT (such as the presentation of evidence); it is the balance and combination of these that is the QRI’s unique contribution. The plan may also recommend further audio-recording of recruitment appointments or documentation of the recruitment pathway, particularly if either aspect has not been completed fully previously, or inclusion of previously optional QRI elements (see above). The implementation of the plan requires close collaboration between the QRI team and the CI of the RCT, and varies depending on the format of the QRI.

### QRI implementation

The QRI is suitable for implementation in two formats:

1. Integrated into the feasibility/pilot or main phase of RCTs for which recruitment is expected to be difficult—aiming to prevent the development of difficulties and optimise recruitment and informed consent, addressing challenges as they arise
2. Applied to RCTs that are underway and showing evidence of serious recruitment shortfalls that threaten the continuation of the RCT–aiming to elucidate opportunities for rapidly increasing recruitment or clearly identifying insurmountable problems

The two phases of the QRI remain in both formats. As the aims are subtly different, the intensity of the research and speed of implementation of the plan will vary. When applied to ongoing RCTs, if there is commitment to facilitate interviewing and audio-recording, phase I can be implemented quickly, and its findings can be produced within 2 months. When phase I is integrated into RCTs in the pilot/feasibility/main stage, the speed of implementation of the QRI will depend on the rate of setting up clinical centres. Implementing in feasibility/pilot studies will often allow findings in an early recruiting centre to be presented to others to encourage optimal recruitment. Training sessions on generic issues can also be provided. The QRI in the feasibility stage of the RCT will also provide detailed data to support the establishment of optimal recruitment practices for the main recruitment stage. The materials developed by the QRI team to provide generic support and training, modified for each RCT in relation to the specific findings, can be adapted as required by the different formats and stages.

### Evaluating the impact of the QRI

The impact of the plan of action can be evaluated in the following ways:

1. The recruitment log and pathway chart can be repeated and compared with earlier versions.
2. Eligibility and randomisation rates can be assessed before the plan of action is implemented and regularly afterwards to check whether these rates improve; it is helpful to look at these by clinical centre and sometimes by individual recruiter.
3. Follow-up interviews can be undertaken with recruiters to ask about the impact and acceptability of the QRI and views about its effectiveness and the changes that occur.
4. Interviews with patients can assess levels of informed decision making.
5. Audio-recording of recruitment appointments post-feedback can be used to investigate changes made and their impact on decision making

The results of evaluations 1 and 2 will need to be interpreted cautiously as they will be observational before-and-after findings, subject to a range of biases including the introduction of the wide range of other things that CIs do to improve recruitment [30]. For ongoing RCTs, the findings are likely to be clearer as there will be a relatively short phase I period and a summary of findings. In a feasibility or pilot study, the findings from the integrated QRI may be produced after relatively short periods in particular clinical centres, as the aim is to improve recruitment over time, in an iterative manner.

The findings from the QRI can be reviewed with the CI/CTU/TMG to decide how to address further needs. The QRI team can provide evidence about the source of recruitment difficulties and offer to provide feedback and facilitate discussion, but it remains the decision of the CI and the commitment of the TMG to enable such feedback and discussion to occur effectively. The CI, CTU, and TMG members play an essential role in putting changes into practice and encouraging others. Decisions can then be made about the need for further feedback and training sessions, reviews of particular clinical centres, presentations to groups of recruiters, or provision of ‘tips for recruiter’ documents, or, if changes are substantial, a re-launch of the RCT with an adjusted protocol and/or a re-drafted PIS. Such events can be plotted on the recruitment chart (see [19] p. 31).

### Examples of the implementation of each format of the QRI in practice

To date, QRIs have been applied in 13 RCTs by the QuinteT team (http://www.bristol.ac.uk/social-community-medicine/research/groups/social-sciences-health/quintet/qri-rcts/). An example of the implementation of each of the two formats is provided below to illustrate how the QRI works in practice:

1. QRI in a feasibility study: the NIHR OPTIMA RCT
  The QRI was established because recruitment was anticipated to be difficult. The NIHR OPTIMA ‘Prelim’ feasibility and pilot study investigated whether it was possible to recruit patients with primary hormone-sensitive breast cancer, who were at high risk of relapse by virtue of regional lymph node metastases or tumour size, to a trial of biomarker-directed treatment. Participants either received chemotherapy followed by hormone therapy treatment immediately (as per current practice) or had treatment determined following a test that had showed promise in indicating whether tumours would be responsive to chemotherapy [31]. Patients randomised to receive the test would receive standard treatment if the test indicated it was needed or hormone therapy only if the test indicated chemotherapy was not required. The CI and TMG were concerned that patients would not be willing to forego chemotherapy or be prepared to wait for a test result, so a QRI was embedded in the feasibility phase [31].
  The QRI followed the phases and stages as indicated above. Interviews with recruiting oncologists (*n* = 14) revealed several ‘hidden challenges’ [31]. Although recruiters expressed clear support and enthusiasm for the RCT, at times, they also exhibited discomfort about the eligibility criteria and hesitancy about presenting the RCT to patients who, although they were eligible for the RCT according to the protocol, had clinical or other characteristics that they thought should or should not require chemotherapy. Analysis of audio-recorded appointments (*n* = 36) revealed that recruiters mostly provided clear and detailed diagnostic information but sometimes found it difficult to suspend routine practices that contradicted the RCT’s premise, for example, unwittingly recommending chemotherapy. Some recruiters also found it difficult to explain the RCT design and treatment allocation in lay terms, particularly as in the OPTIMA RCT, the ‘test-directed’ treatment arm was divided into two distinct allocations, depending on the result of the test. Review of the first study PIS identified several occasions where explanations of the treatment allocation had the potential to lead to confusion.
  The QRI plan of action agreed on by the CI and TMG for the OPTIMA RCT involved drafting and dissemination of ‘tips and guidance’ sheets to recruiters, amendments to the PIS, and a series of individual and group feedback sessions. Tips and guidance sheets provided recommendations on how to introduce the study and explain the trial design, with similar approaches taken to clarify the information provided in the PIS. More subtle difficulties such as issues of equipoise around eligibility criteria and problematic terminology were discussed in four regional group feedback sessions attended by oncologists, research nurses, members of the TMG, and the CI. Clinical vignettes displaying patients at the extremes of eligibility criteria were displayed and discussed to air discrepancies in opinion and sources of discomfort. More delicate issues of managing ‘gut instincts’ when approaching patients were discussed through confidential individual feedback sessions with recruiting oncologists (*n* = 4).
  Full details of the QRI integrated in the feasibility ‘prelim’ study and how the recruitment targets were reached have now been published [31]. The NIHR OPTIMA RCT has now been funded for full-scale recruitment, with a further integrated QRI to optimise recruitment. Findings from the QRI are being used to inform training and further drafts of guidance for recruiters to be implemented in the large number of new centres in the full-scale RCT.
2. QRI in an ongoing RCT with recruitment difficulties: the ARUK CSAW trial
  The CSAW RCT aimed to investigate the effectiveness of treatment for subacromial pain in the shoulder by comparing active surgery (arthroscopic subacromial decompression), an investigational shoulder arthroscopy without surgery, and a non-operative option with monitoring and specialist assessment [32]. The research included an opportunity for patients who did not want to join the RCT to participate in a ‘preference’ study (observational follow-up of patients indicating a preference for a particular treatment). The QRI was established after recruitment had been undertaken for 8 months but was falling behind the target figure, leading the funder ARUK (Arthritis Research UK) to issue an alert expressing concern.
  The applied QRI broadly followed the phases and stages as indicated above. Interviews were held with four members of the TMG and 13 recruiters from six clinical centres. Logs documenting eligibility and recruitment were monitored on an ongoing basis, and patient pathways were tracked from each of the six centres. Recruitment appointments (*n* = 25) were recorded in four centres. The recruitment difficulties included both clear obstacles and hidden challenges. The most important issue was a lack of equipoise among recruiters. This led to hesitant presentation of the RCT to patients, particularly those who, although eligible for the RCT, were thought to ‘need’ surgery. A lack of consistency and balance existed in how the treatment arms were described, with difficulties particularly in relation to explaining the monitoring option. The existence of the ‘preference’ study meant that recruiters could readily accept patient preferences without exploration, and this acted to discourage participation in the RCT.
  The findings from the QRI were fed back to the CIs and members of the core CSAW RCT team after 4 months. The presentation of the anonymised findings provoked a detailed discussion about the equipoise issues, options for the clearer presentation of the RCT, and the QRI team’s suggestion to close the preference study. This was the first part of the plan of action, which also included personalised feedback, the inclusion of recruitment ‘tips’ in the RCT newsletter, and a training day attended by more than 40 recruiters from 15 centres during which the QRI findings were presented and proposed improvements discussed. In addition, there were five ‘trouble-shooting’ visits to centres that had found recruitment particularly difficult, and a guidance document on how to structure recruitment appointments was sent to all participating centres; this document was based on the QRI findings and the discussions from the training day. The RCT completed recruitment after 35 months, and the findings are awaited [32].

## Discussion

A recruitment intervention (the QRI) has been developed to address recruitment challenges, and has been applied successfully in 13 RCTs to date. ‘Success’ is achieved either by optimising practices that enable recruitment to be completed in feasibility/pilot or main RCTs, or providing detailed evidence to support a decision to cease recruitment. A major aim of a QRI is to understand the recruitment process as it happens and to address clear obstacles and hidden challenges [12] to the provision of clear and accurate information to facilitate informed decision-making by patients about RCT participation. The QRI uses standard and innovative research methods (primarily qualitative and some simple quantification) to provide a clear understanding of the application of the RCT protocol in clinical settings. The first phase of the QRI investigates eligibility assessment and recruitment from the perspective of RCT designers and recruiters in interviews as well as the recruitment process as it actually happens through data collected in logs, and also how the RCT is presented to potential participants in study documents and audio-recordings of appointments. In phase II, anonymised feedback of the phase I QRI findings to the CI/CTU/TMG enables the development of a specifically tailored ‘plan of action’ to improve recruitment and informed consent. A key part of the plan is anonymised feedback, based on the findings, to groups of recruiters, to particular clinical centres, or confidentially to individual recruiters. This feedback encourages reflection about the emotional and intellectual challenges that recruiters (doctors or nurses) often experience, particularly in relation to equipoise, patient eligibility, and perceived role-conflict [13]. Another important aspect of the plan of action is to ensure that information about the RCT is presented as clearly and understandably as possible to increase patient understanding of the rationale of the RCT and the practicalities of research participation. The main aim is to encourage greater patient and recruiter engagement in decision making—with evidence from audio-recordings of participation in appointments—as well as written informed consent [25]. A description of the Phases that constitute the QRI and two examples of its application in two different RCT contexts provide guides to its use more widely.

Recruitment is frequently identified as a major problem for RCTs [2, 4]. Interventions that lead to more effective and efficient recruitment are urgently required, particularly as so many systematic reviews have identified so few [6, 33]. The small number of effective interventions that have been identified have tended to focus on simple administrative aspects aimed at RCT participants, such as ways to increase questionnaire responses [6]. Few have supported recruiters, although there is evidence of demand for these [5, 14]. Most interventions have been developed to address issues identified within particular RCTs, as CIs and RCT staff attempt to do everything they can to rectify poor recruitment. This limits the generalisability of interventions and deals only with issues that are clearly evident to CIs and RCT staff (not the ‘hidden’ challenges [13]).

The QRI was developed to address the limitations with existing interventions in three ways. First, it was developed to understand the recruitment process as it occurred in clinical centres and from the perspectives of recruiters and patients—through the use primarily of qualitative research methods. Second, during its development period, it was applied in as many different sorts of RCTs as possible. Third, it sought to address the difficulties that arose for recruiters and potential participants by actively optimising recruitment quality and informed consent.

In terms of its development using qualitative methods, the QRI built on pioneering research that was conducted alongside pragmatic RCTs (or integrated within them) and produced important incremental insights about understanding experiences of RCT participation. Several groups illuminated, for example, the difficulties for patients arising from poor explanations of randomisation [9, 10] and the importance of clear presentation of equipoise [34, 35]. A small number of studies explored the experiences of clinician recruiters [14, 36, 37]. More recently, a synthesis of interviews conducted with recruiters during the application of the developmental version of the QRI in six RCTs led to a detailed and nuanced understanding of recruitment as a complex and fragile process, fraught with logistical/organisational obstacles, and the identification of several previously hidden emotional and intellectual challenges for recruiters [12, 13].

The application of the QRI in a wide range of different RCTs in terms of their designs, clinical contexts, and recruitment processes provided opportunities to identify generic as well as RCT-specific outcomes. While there were specific recruitment issues in most of the RCTs that were of interest to specialists, [21–23], there were also findings of more generic concern that fed into the further development of the QRI [8]. The final version of the QRI emerged from the symbiotic relationship between the insights gained from the qualitative research about recruitment from the perspectives of patient participants and clinician/nurse recruiters and the implementation of the plan by recruiters to improve recruitment based on those understandings. The theoretical background justifying this approach originates from the view that RCTs are Complex Adaptive Systems (CAS)—collections of individual agents (recruiters) with the freedom to act in ways that are not always predictable [38]. There is a need with CAS to understand the contextual factors influencing behaviour (QRI-determined clear obstacles and hidden challenges), with built-in feedback loops to enable changes to agents’ actions (the QRI plan of action).

The necessity of integrating the QRI in the RCT reflects its holistic approach to optimising recruitment and the need to engage patients and recruiters in the process. Its aim is to ensure that the presentation of the RCT is accurate and fair, so that recruiters and patients can fully discuss the purpose of the research, and patients can make informed decisions about whether to take part. The neglected aspect in many previous interventions has been the lack of understanding about recruiters’ views and instincts about the RCT. Clinicians need to be ‘in equipoise’ to believe that there is no advantage or disadvantage for a patient to receive one of the RCTs arms [39] and to be uncertain about the best option because of the lack of evidence [40]. There have been many debates about the philosophical issues underlying equipoise, but now it has also been shown empirically that it raises immense difficulties for some recruiters, and that they may need support and training to deal with it [13, 14, 36]. Further, if these ‘hidden’ issues are not openly discussed, the presentation of the RCT to patients is likely to be affected and the participation and decision making of the patients may be compromised [12]. The feedback process in the QRI encourages recruiters to reflect on factors such as their own understanding of the evidence, their own equipoise, the balance between community equipoise with colleagues and in relation to actual patients, and the potential conflicts that can emerge from combined clinical and research roles [12].

For patients (potential RCT participants), the provision of clear information is crucial for fully informed consent. Ethics and governance procedures should ensure this, but they focus primarily on the written PIS. Audio-recording recruitment appointments in the QRI has shown that these interactions are crucial for enabling patient engagement with difficult aspects of RCT participation such as preferences [27]. Ultimately, full engagement of patients as potential participants will be beneficial for recruitment to RCTs. When clinicians and other recruiters, as well as patients, perceive that participating in the RCT is an appropriate decision in the face of uncertainty regarding the optimal treatment, a more engaged and participatory form of informed consent can be achieved. This is the primary aim of the QRI, and if there is no confidence in the RCT, recruitment should not continue (see, for example, [22]).

The costs of a QRI primarily relate to the need to employ an experienced qualitative researcher at 0.5 FTE for the first year of recruitment (with associated on-costs and overheads, this amounts to c.£60–80,000). A formal cost-effectiveness analysis has not yet been undertaken, but if the QRI is effective in increasing recruitment, the costs of the main RCT are likely to be reduced.

### Strengths and limitations of the QRI

The strength of the QRI is its grounding in a nuanced understanding of the recruitment process based on its own and others’ qualitative research, its focus on the needs of recruiters and potential participants, and its application in RCTs that anticipate recruitment challenges, or are underway and experiencing difficulties. It is limited, however, by the availability of only observational evidence of its effectiveness and not yet proven cost-effectiveness. Undertaking a randomised study to robustly evaluate it is a goal, but this is not without severe difficulties. An RCT of QRIs applied to feasibility or pilot RCTs would be particularly difficult, as the iterative nature of the data collection and spread of the influence of feedback and training across centres produces an accumulation of evidence rather than enabling analysis of a static primary outcome. QRIs applied to ongoing RCTs with recruitment shortfalls might be possible as there is then a more rapid data collection period and a clearer implementation phase. But there are then questions about whether to randomise clinical centres or whole RCTs, which would present practical and methodological challenges unless a sizable funding body agreed to randomise its portfolio of RCTs with recruitment problems. There would remain, however, the thorny issue of what the outcome measure would be. Simple quantitative measures of eligibility and recruitment rates could be used, but they are only part of the story. Of greater importance is the quality of the recruitment as represented by evidence of truly engaged and informed consent by participants. Such a measure is currently under development in QuinteT as existing measures focus primarily on recruiter information provision [41, 42].

Other aspects that remain to be evaluated include levels of compliance with the randomised allocation and whether the QRI leads to greater or lesser longer-term retention. Another potential limitation is that QRIs have only been implemented by one team—QuinteT (http://www.bristol.ac.uk/social-community-medicine/research/groups/social-sciences-health/quintet/qri-rcts/)—although several CIs, CTUs and TMGs have been involved. This paper is aimed at enabling others to understand the basis and methods, so the intervention can be spread in collaboration with the QuinteT team and/or implemented more widely. It is important to note that many of the qualitative methods underlying the QRI are standard: in-depth interviews, content, and thematic approaches to analysis using the techniques of constant comparison, for example. The QuinteT team has also developed several innovative methods to address particular issues, such as Q-QAT to assess the balance of information provision [23]. Other aspects, however, will be unfamiliar to traditional qualitative researchers, including the use of targeted methods of data collection and analysis that are essential to produce findings quickly while retaining robustness. Another unfamiliarity is the intention of the QRI at the outset to influence the conduct of the recruitment process of the RCT and put into place opportunities for discussion and feedback that may lead to changes to RCT recruitment, overall conduct, or even design. The QuinteT team has developed working methods and team approaches to support the research; it will be interesting to see how much of this is transferable.

## Conclusion

The QRI provides a flexible way of understanding recruitment difficulties and producing a plan of action to address them and ensure engaged and well-informed decision making by patients. A QRI integrated into an RCT at the feasibility/pilot/main trial stage can be used to elicit recruitment challenges with a view to preventing them or addressing them as they arise. Used in this way, the QRI can ensure that recruitment to even the most difficult and controversial (but important) RCTs can be attempted. Where recruitment difficulties have developed unexpectedly, the QRI can be applied to the RCT to identify the sources of the difficulties and provide evidence to improve recruitment or discontinue it. QRIs are likely to be of interest to CIs and CTUs developing proposals for ‘difficult’ RCTs and for those RCTs with lower than expected recruitment. In addition, while it has not been formally studied, QRIs should lead to more efficient completion of RCTs with enhanced patient participation, which should be of interest to funding bodies.

## Abbreviations

CI, chief investigator; Q-QAT, quanti-qualitative appointment timing; QRI, QuinteT Recruitment Intervention; RCT, randomised controlled trial

## Additional file

Additional file 1: Contains details of ethical approvals for the RCTs and ProtecT trial registrations. (DOCX 45 kb)
