# Supplementary material for: Optimising recruitment and informed consent in randomised controlled trials: the development and implementation of the Quintet Recruitment Intervention (QRI)
Source: Trials. 2016 Jun 8;17:283. doi: 10.1186/s13063-016-1391-4 (PMC4898358; doi:10.1186/s13063-016-1391-4)
Supplement: Additional file 1: — Contains details of ethical approvals for the RCTs and ProtecT trial registrations. (DOCX 45 kb) [file 13063_2016_1391_MOESM1_ESM.docx]

Approvals for research in the RCTs for the development of the QRI were through the ProtecT study, by Trent Multicentre Research Ethics Committee (ref: 01/4/025), the MRC Quartet study by the NHS Research Ethics Committee, Leeds West (07/02/2005 ref: 04/Q1205/179), and through the SPARE RCT, by South East Research Ethics Committee (ref: 06/MRE01/95). Approval for the Optima Prelim study was given by the South East Coast Surrey Research Ethics Committee (22/06/12, Ref: 12/LO/0515), and for CSAW by the NHS Research Ethics Service, South Central-Oxford B (02/02/2012 ref: 12/SC/0028). The ProtecT trial was registered as ISRCTN20141297 and NCT02044172.
